# Increased costs associated with greater adherence to the EAT-Lancet Commission reference diet in the province of Québec: the PREDISE Study

**DOI:** 10.1017/S0007114525000364

**Published:** 2025-03-28

**Authors:** Gabrielle Rochefort, Marie-Claude Paquette, Julie Robitaille, Simone Lemieux, Véronique Provencher, Benoît Lamarche

**Affiliations:** 1 Centre Nutrition, santé et société (NUTRISS), Institut sur la nutrition et les aliments fonctionnels (INAF), Université Laval, Québec QC G1V 0A6, Canada; 2 École de nutrition, Faculté des sciences de l’agriculture et de l’alimentation, Université Laval, Québec, QC G1V 0A6, Canada; 3 Institut national de santé publique du Québec, Montréal, QC H2P 1E2, Canada

**Keywords:** EAT-Lancet diet, EAT-Lancet dietary index, Diet cost, Affordability, Food price, Sustainability, Sustainable diet

## Abstract

The diet proposed by the EAT-Lancet Commission has faced criticism concerning its affordability. This study aimed to investigate the cost associated with a greater alignment to the EAT-Lancet reference diet in the province of Québec, Canada. The dietary habits of 1147 French-speaking adults were assessed using repeated web-based 24-h recall data collected between 2015 and 2017 in the cross-sectional PRÉDicteurs Individuels, Sociaux et Environnementaux (PREDISE) study. Diet costs were calculated using a Nielsen food price database. Usual dietary intakes and diet costs were estimated using the National Cancer Institute’s multivariate Markov Chain Monte Carlo method. Adherence to the EAT-Lancet diet was assessed using the EAT-Lancet dietary index (EAT-I). Associations between diet costs and EAT-I scores were evaluated using linear regression models with restricted cubic splines. After adjustment for energy intake, a higher EAT-I score (75th *v*. 25th percentiles) was associated with a 1·0 $CAD increase in daily diet costs (95 % CI, 0·7, 1·3). This increase in diet costs was mostly driven by the following component scores of the EAT-I (75th *v*. 25th percentiles, higher scores reflecting greater adherence): vegetables (1·6 $CAD/d, 95 % CI: 1·2, 2·1), free sugars (1·6 $CAD/d, 95 % CI: 1·3, 1·9), fish and plant-based proteins (1·4 $CAD/d, 95 % CI: 1·0, 1·8), fruits (0·9 $CAD/d, 95 % CI: 0·4, 1·3) and whole grains (0·4 $CAD/d, 95 % CI: 0·0, 0·8). Inversely, a greater score for the poultry and eggs component was associated with reduced diet costs (–1·2 $CAD/d, 95 % CI: −1·7, −0·7). This study suggests that adhering to the EAT-Lancet diet may be associated with an increase in diet costs in the province of Québec.

The current global food system is one of the main drivers of environmental degradation due to unfavourable impacts on global warming^(1)^, eutrophication^(2)^, freshwater use^(3)^, land use and biodiversity^(4)^. Coupled with the deleterious effects of current dietary patterns on morbidity and mortality^(5–7)^, this provides strong arguments to help the population shift to more sustainable dietary patterns. Based on the definition of the FAO of the UN, the 2016 Chicago Consensus on Sustainable Food Systems Science proposed that sustainable dietary patterns encompass four domains, namely, health, environment, economics and society^(8)^. These patterns should be ‘protective and respectful of biodiversity and ecosystems, culturally acceptable, accessible, economically fair and affordable, nutritionally adequate, safe and healthy, while optimising natural and human resources’^(9)^. Along those lines, the EAT-Lancet Commission on healthy diets from sustainable food systems proposed in 2019 a reference dietary pattern aimed at providing adequate nutrition to the world population while remaining within planetary boundaries^(10)^.

Providing affordable and nutritionally adequate diets without threatening the environment represents one of the greatest challenges of our time. It is well established that adhering to healthier dietary patterns increases daily diet costs^(11–18)^ as nutritious foods such as fruits and vegetables generally cost more per calorie than energy-dense and low-nutrient highly processed foods such as refined grains, snacks and sweets that are often high in sodium, sugars or saturated fats^(19–22)^. Dietary patterns characterised by lower greenhouse gas emissions have also recently been found to cost more^(23)^, reflecting the many challenges in achieving the best trade-offs for sustainable yet economically accessible diets.

Concerns about the cost and affordability of the EAT-Lancet dietary pattern have been raised, and the EAT-Lancet Commission has been criticised for largely disregarding the economic aspect of the proposed reference diet^(24)^. A recent study showed that the EAT-Lancet reference diet was not affordable for about 1·6 billion people around the world^(25)^. Given that cost is a fundamental determinant of dietary choices^(26,27)^ and that the EAT-Lancet reference diet represents a benchmark for healthy and sustainable dietary patterns, documenting its cost in different countries is crucial to inform future policies on sustainable eating. Thus, this study aimed to assess the costs associated with greater adherence to the EAT-Lancet reference dietary pattern in the province of Québec, Canada. We hypothesise that greater adherence to the EAT-Lancet dietary pattern is associated with greater daily diet costs.

## Methods

### Study population

Data used for these analyses were from the web-based multicenter cross-sectional PRÉDicteurs Individuels, Sociaux et Environnementaux (PREDISE) study^(28)^. In short, the PREDISE study aimed to document associations between individual, social and environmental factors and adherence to dietary guidelines. Recruitment took place between August 2015 and April 2017 and aimed for a final sample size of 1000 French-speaking adults aged between 18 and 65 years from five administrative regions of the province of Québec (i.e. Capitale-Nationale/Chaudière-Appalaches, Estrie, Mauricie, Montreal and Saguenay-Lac-St-Jean). Recruitment was completed through a survey firm using a stratified sampling design to ensure sex and age representativeness in each administrative region. To be included, participants had to have Internet access to complete the questionnaires. Exclusion criteria included being pregnant or lactating and intestinal malabsorption as these conditions are associated with important transitional changes in dietary habits and could also impact fasting blood assessments taken as part of the PREDISE study protocol. Sociodemographic and dietary intake data were collected over a 3-week period. In total, 1849 participants met the inclusion criteria, and 1147 of them, whose dietary intake data were collected, were included in the present study. The PREDISE study was conducted according to the guidelines laid down in the Declaration of Helsinki, and all procedures involving participants were approved by the Research Ethics Committees of *Université Laval* (ethics no. 2014-271), *Centre hospitalier universitaire de Sherbrooke* (ethics no. MP-31-2015-997), Montreal Clinical Research Institute (ethics no. 2015-02) and *Université du Québec à Trois-Rivières* (ethics no. 15-2009-07.13). Written informed consent was obtained from all participants.

### Dietary intake assessment

Participants’ dietary intakes were assessed using three unannounced web-based 24-h dietary recalls (R24W) completed over 3 weeks. Complete procedures regarding the development and validation of the R24W have been described previously^(29,30)^. In the R24W, mixed dishes are broken down into individual foods of the Canadian Nutrient File (v2015), which is used to generate nutrient values. Each food reported in the R24W is also linked to a Bureau of Nutritional Science (BNS) food group (*n* 181, e.g. 1A-Pasta, 1B-Rice, 2A-White Bread, 10A-Milk whole, 40B-Apple) of the 2015 Canadian Nutrient File.

### Diet costs

Daily diet costs were calculated by linking 24-h dietary recall data to a food price database developed by our research team in collaboration with the *Institut national de santé publique du Québec*. Complete procedures regarding the development of this food price database have also been previously described^(18)^. In brief, a price was calculated for each BNS food group of the R24W using a food price database from Nielsen, which covered the 2015–2016 fiscal year. This food price database included annual sales data in Canadian dollars and kilograms from the three largest grocery chains in Québec (Loblaw, Sobeys, Metro), as well as from three large surface stores (Walmart, Target, Zellers). The total sales (dollars) of the different foods within each of the BNS food groups was divided by the corresponding amount of each food sold (kg) within that group. The estimated price of each BNS food group, calculated by averaging the prices of all foods within each BNS food group, was therefore weighed for the volume of sales of each food. The Nielsen database did not provide food prices for 47 of the 181 BNS food groups. The different approaches used to obtain food prices in such instances are detailed in the online Supplementary Table 1. Food prices were standardised for material loss and food preparation (i.e. moisture, fat loss and cooking gains). The amount of each food or beverage reported in the R24W and expressed in kilograms was multiplied by the corresponding BNS food group price per kilogram. The cost of each food and beverage reported was added up to obtain a daily diet cost for each 24-h dietary recall completed by participants.

### EAT-Lancet dietary index

Data from 24-h dietary recalls were used to calculate the EAT-Lancet dietary index (EAT-I), which assesses the alignment of dietary patterns with the EAT-Lancet reference diet. Complete methods on the development and validation of this index have been described elsewhere^(31)^. The EAT-I consists of ten main components: (1) whole grains, (2) tubers and starchy vegetables, (3) vegetables, (4) fruits, (5) dairy foods, (6) red and processed meats, (7) poultry and eggs (poultry and eggs subcomponents), (8) fish and plant-based proteins (fish, legumes and nuts subcomponents), (9) added fats (saturated fats and unsaturated fats subcomponents) and (10) free sugars. The whole grains, vegetables, fruits, fish and plant-based proteins components and unsaturated fats subcomponent are considered as adequacy components or subcomponents for which higher consumption results in higher adherence scores. The tubers and starchy vegetables, red and processed meats, free sugars components and saturated fats subcomponent are defined as moderation components or subcomponents for which a lower consumption results in higher adherence scores. Finally, the dairy foods and poultry and eggs components are defined as optional components for which adherence scores vary within the distribution of intakes. All main components of the EAT-Lancet dietary pattern were scored on a 10-point scale, except the dairy foods, red and processed meats, poultry and eggs and fish and plant-based proteins components, which were each scored on a 5-point scale. The EAT-I total score ranges from 0 to a maximum of 80 points (see online Supplementary Table 2 for the EAT-I components, points and scoring system).

### Statistical analyses

To take the stratified sampling design of the PREDISE study into account, SURVEY procedures were used when applicable (see below). Balancing weights were applied to ensure sex and age representativeness in each administrative region, consistent with the study’s initial design, as the final study sample size was larger than originally planned. Missing sociodemographic characteristics (i.e. household income (*n* 159) and education (*n* 60)) were imputed once using the fully conditional specification method. SURVEYMEANS procedures were used to calculate the mean energy-adjusted daily diet costs (i.e. /2500 kcal) in the entire sample and subgroups based on sex (men, women), age (18–34 years, 35–49 years, 50–65 years), education (none, high school, trade diploma/Collège d’Enseignement Général et Professionnel/university), household income (< 30 000 $CAD, 30 000 to < 60 000 $CAD, 60 000 to < 90 000 $CAD, ≥ 90 000 $CAD), smoking status (smokers, non-smokers) and administrative region of residence (Capitale-Nationale/Chaudière-Appalaches, Estrie, Mauricie, Montreal and Saguenay-Lac-St-Jean). The mean EAT-I score and component scores in the entire sample and subgroups were estimated with the National Cancer Institute (NCI) population ratio method, and 95 % CI were estimated using 200 bootstrap resamples^(32)^.

Self-reported energy intakes from all 24-h dietary recalls were assessed for plausibility by comparing them with predicted energy requirements using the approach by Huang *et al.* ^(33)^. The equations for predicting energy requirements assumed a lightly active physical activity level for all participants. The within-individual CV for total energy intakes was determined using the NCI univariate method with log-transformed data. The biological variability in total energy expenditure was obtained from Black and Cole^(34)^.

Distributions of usual food and nutrient intakes and of usual daily diet costs were estimated with the NCI multivariate Markov Chain Monte Carlo method^(35)^ using data from all completed 24-h dietary recalls. This method uses regression calibration to account for within-individual random errors measured with 24-h dietary recalls that affect dietary intake data, including diet costs. The model used for the present analyses was stratified by sex to better reflect within-individual random dietary intake variations and included the following covariables: indicators for the sequence of 24-h recalls (i.e. first, second or third recall) and the day of the week (i.e. weekdays *v*. weekend days including Friday)^(36)^, age, smoking status, household income, education and administrative region. The following foods were considered episodic variables in the model because 10 % or more of participants did not report consumption on their first R24W: whole grains, starchy vegetables, vegetables, fruits, red and processed meats, poultry, eggs, fish and seafood, legumes and soy, nuts and seeds, saturated added fats and unsaturated added fats. The remaining foods and nutrients, including energy as well as diet costs, were considered as daily variables. For a pre-specified number of pseudo-individuals per participant (i.e. 500 simulations), usual dietary intakes and costs were generated during the Monte Carlo simulation step and pooled within each sex stratum. The EAT-I score and its component scores were calculated from estimated usual intakes among pseudo-individuals.

Linear regression models with restricted cubic splines were used to evaluate associations between diet costs (dependent variable) and the EAT-I score and its component scores (independent variables) in the overall sample and predetermined subgroups. Knots at the 5th, 35th, 65th and 95th percentiles were used for the following independent variables: EAT-I score, whole grains, poultry and eggs, fish and plant-based proteins, added fats and free sugars components. For the tubers and starchy vegetables, vegetables and fruits components, knots at the 10th, 50th and 90th percentiles were used to account for their skewed distribution. A linear regression model without restricted cubic splines was used for the dairy foods component as there was not enough variation in its distribution. Finally, it was not possible to evaluate the association between daily diet costs and the red and processed meats component of the EAT-I because most participants received the same number of points. Differences in daily diet costs between the 75th and 25th percentile of the EAT-I score and component score distributions were calculated to estimate the effect size. Given that diet cost is closely linked to the amount of calories consumed, analyses were performed with and without adjustment for energy intake. Standard errors and 95 % CI were estimated using 200 bootstrap resamples. All analyses were performed in SAS Studio (SAS Institute), and figures were generated in R studio (R Foundation for Statistical Computing).

## Results

As shown in Table 1, 50·2 % of participants were women, 44·9 % had a university degree and 34·0 % had a total annual household income of ≥ 90 000 $CAD. The estimated mean EAT-I score was 33·4/80 points (95 % CI, 32·2, 34·6), and energy-adjusted daily diet costs were 12·7 $CAD/2500 kcal (95 % CI, 12·5, 12·9). Females, participants aged 50–65 years, participants with a university degree and non-smokers had a higher estimated mean EAT-I score than their counterparts. Energy-adjusted daily diet costs were higher among participants aged 50–65 years and smokers than their counterparts. The extent of potential under- and over-reporting of total energy intake was 20·7 and 14·9 % (*n* 1022), respectively (data not shown).

**Table 1.** Participants’ sociodemographic characteristics, EAT-I scores and energy-adjusted daily diet costs (Numbers and percentages; means and 95 % CI)

	Participants		EAT-I scores^*^	95 % CI	Energy-adjusted diet costs, $CAD/d^†^	95 % CI
	*n*	%	Means, 95 % CI		Means, 95 % CI	
	1147	100	33·4	32·2, 34·6	12·7	12·5, 12·9
Sex						
Women	576	50·2	37·6	35·9, 39·3	12·8	12·5, 13·0
Men	571	49·8	30·5	29·1, 31·9	12·7	12·4, 13·0
Age						
18–34 years	408	35·6	31·7	30·0, 33·4	12·0	11·7, 12·3
35–49 years	338	29·5	33·5	31·4, 35·5	12·7	12·3, 13·1
50–65 years	400	34·9	36·2	34·2, 38·2	13·5	13·1, 13·8
Education^‡^						
None, high school or trade diploma	281	24·4	29·7	27·3, 32·2	12·5	12·0, 12·9
CEGEP	352	30·7	31·7	30·2, 33·1	12·9	12·5, 13·2
University	514	44·9	37·5	35·6, 39·4	12·8	12·5, 13·0
Household income^‡^						
< 30 000 $CAD	207	18·0	31·7	28·9, 34·4	11·9	11·4, 12·4
30 000 to < 60 000 $CAD	329	28·7	32·6	30·3, 34·9	12·9	12·5, 13·3
60 000 to < 90 000 $CAD	221	19·3	34·1	31·5, 36·7	12·7	12·3, 13·1
≥ 90 000 $CAD	390	34·0	34·6	32·8, 36·5	13·1	12·7, 13·4
Smoking status						
Non-smokers	984	85·8	34·7	33·4, 35·9	12·6	12·4, 12·8
Smokers	163	14·2	26·7	24·4, 28·9	13·5	12·8, 14·2
Administrative region						
Capitale-Nationale/Chaudière-Appalaches	435	37·9	33·2	31·5, 34·8	13·0	12·7, 13·4
Estrie	110	9·6	34·6	31·5, 37·6	12·6	12·1, 13·1
Mauricie	99	8·6	30·5	26·9, 34·2	13·3	12·6, 14·1
Montreal	397	34·6	36·3	34·2, 38·4	12·4	12·1, 12·7
Saguenay-Lac St-Jean	107	9·3	28·3	25·3, 31·3	12·4	11·7, 13·0

EAT-I, EAT-Lancet dietary index; CAD, Canadian dollars; CEGEP, Collège d’Enseignement Général et Professionnel.

* EAT-I scores estimated with the National Cancer Institute population ratio method. 95 % CI estimated using 200 bootstrap resamples.

† Energy-adjusted diet costs reported for 2500 kcal. Mean energy-adjusted diet costs not estimated with the National Cancer Institute multivariate method.

‡ Missing sociodemographic characteristics imputed (see Methods section).

**Figure 1.**
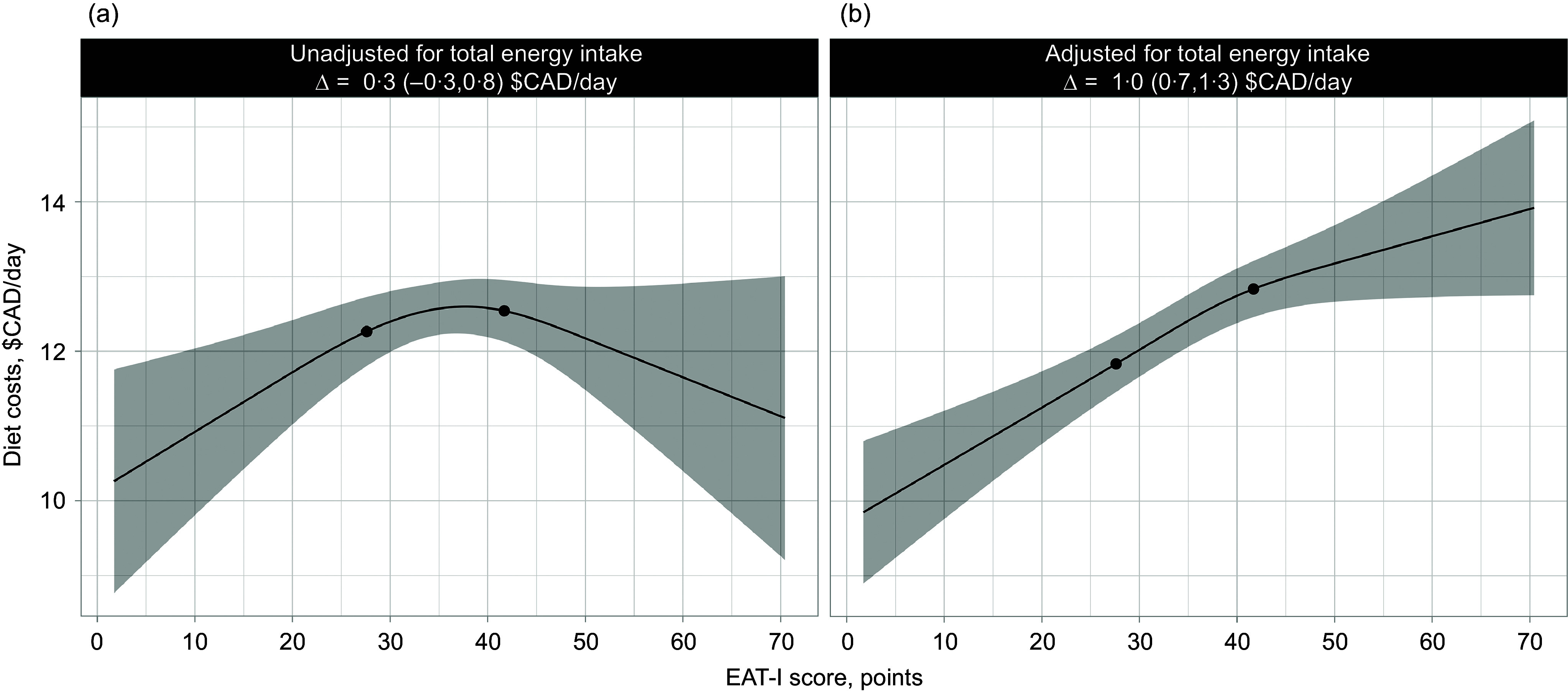
Linear regressions of EAT-I scores and daily diet costs in 1147 French-speaking adults from Quebec. A higher EAT-I score indicates a stronger agreement with the EAT-Lancet reference diet. The black dots on the regression line represent the 25th and 75th percentiles of the EAT-I score distribution. The estimates presented correspond to the daily diet costs difference (∆) between the 75th and 25th percentiles of the EAT-I score distribution. The shaded area represents the 95 % CI of the regression. (a) Linear regression of EAT-I scores and daily diet costs with no adjustment for energy intake. (b) Linear regression of EAT-I scores and daily diet costs adjusted for energy intake. Usual diet costs and dietary intakes were estimated with the National Cancer Institute multivariate Markov Chain Monte Carlo method, and 95 % CI were obtained using 200 bootstrap resamples. CAD, Canadian dollars; EAT-I, EAT-Lancet dietary index.

**Figure 2.**
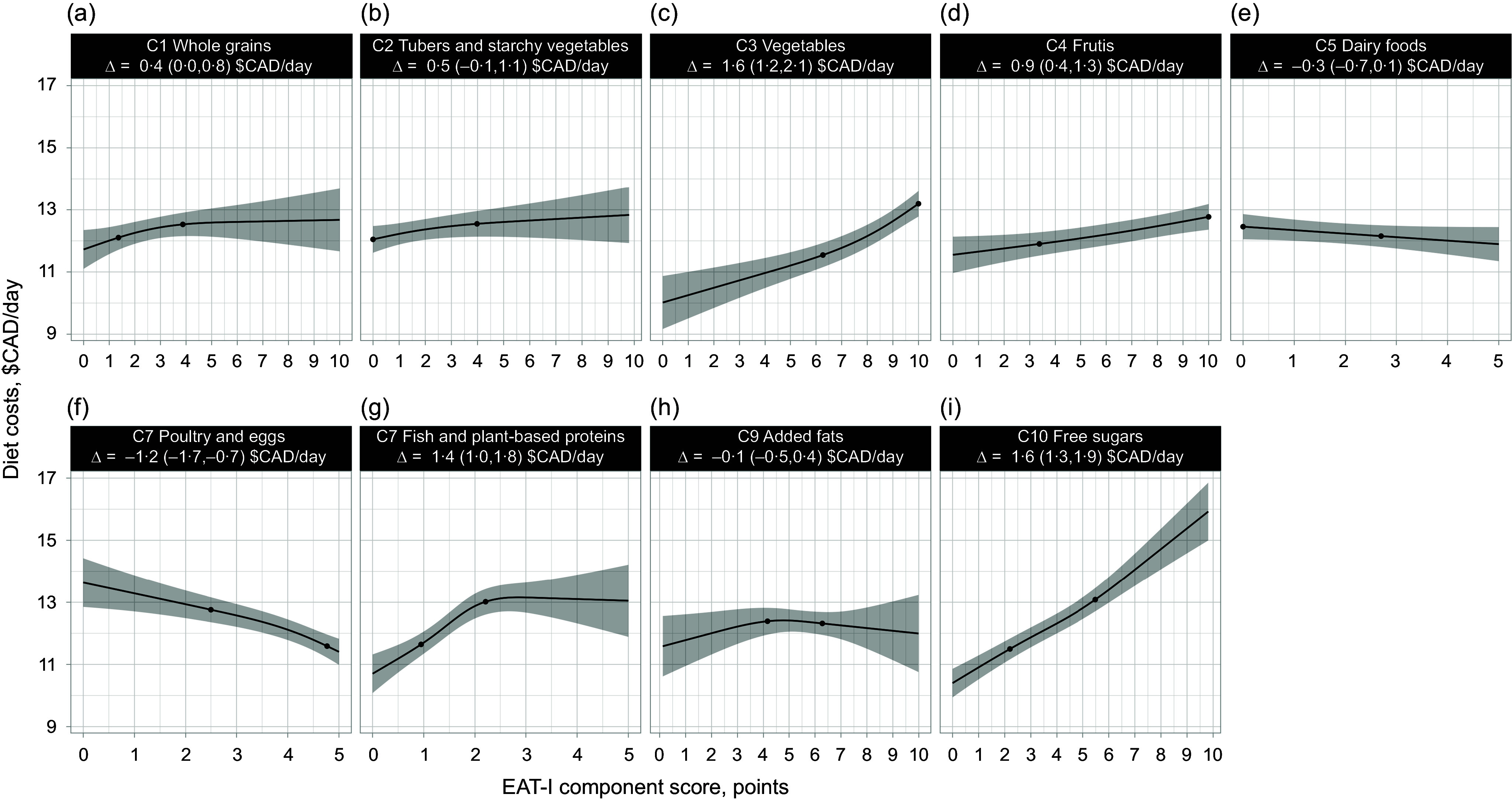
Linear regressions of EAT-I component scores and daily diet costs adjusted for total energy intake in 1147 French-speaking adults from Quebec. Higher EAT-I component scores indicate a stronger agreement with individual recommendations of the EAT-Lancet reference diet. The black dots on the regression line represent the 25th and 75th percentiles of the EAT-I component score distribution. The estimates presented correspond to the energy-adjusted daily diet costs difference (∆) between the 75th and 25th percentiles of the EAT-I component score distribution. The shaded area represents the 95 % CI of the regression. Usual diet costs and dietary intakes were estimated with the National Cancer Institute multivariate Markov Chain Monte Carlo method, and 95 % CI were obtained using 200 bootstrap resamples. Results are not presented for the red and processed meats component of the EAT-I as it was not possible to calculate the energy-adjusted cost difference between the 75th *v*. 25th percentile of the component score distribution (see Methods section). CAD, Canadian dollars; EAT-I, EAT-Lancet dietary index.

Figure 1 presents linear regressions of daily diet costs and EAT-I scores in 1147 French-speaking adults of the province of Québec. When no adjustment for energy intake was performed, there was no cost difference comparing high (75th percentile) *v*. low (25th percentile) EAT-I scores (0·3 $CAD/d, 95 % CI, −0·3, 0·8). On the other hand, when adjusted for energy intake, there was a 1·0 $CAD/d difference (95 % CI, 0·7, 1·3) in diet costs between high (75th percentile) and low (25th percentile) EAT-I scores. The greater energy-adjusted daily diet costs with increasing EAT-I scores was explained by greater adherence to the following components (Figure 2): whole grains (0·4 $CAD/d, 95 % CI, 0·0, 0·8), vegetables (1·6 $CAD/d, 95 % CI, 1·2, 2·1), fruits (0·9 $CAD/d, 95 % CI, 0·4, 1·3), fish and plant-based proteins (1·4 $CAD/d, 95 % CI, 1·0, 1·8) and free sugars (1·6 $CAD/d, 95 % CI, 1·3, 1·9). In contrast, the poultry and eggs component score of the EAT-I was negatively associated with diet costs (–1·2 $CAD/d, 95 % CI, −1·7, −0·7).

Finally, the daily diet costs difference comparing high (75th percentile) *v*. low (25th percentile) EAT-I scores did not differ among sociodemographic subgroups of individuals based on sex, age, education, household income, smoking status and administrative region of residence, with all sociodemographic subgroups showing a similar increase in daily diet costs (Table 2).

**Table 2.** Differences in daily diet costs between high (75th percentile) and low (25th percentile) EAT-I scores adjusted for total energy intake among sociodemographic subgroups in 1147 French-speaking adults of the province of Québec^*^

	Energy-adjusted diet cost differences, 75th *v*. 25th percentiles of EAT-I scores ($CAD/d)^†^	95 % CI^‡^
Sex		
Women	1·0	0·7, 1·4
Men	1·1	0·6, 1·5
Age		
18–34 years	0·9	0·6, 1·3
35–49 years	1·0	0·6, 1·3
50–65 years	0·8	0·5, 1·2
Education^§^		
None, high school or trade diploma	1·0	0·6, 1·5
CEGEP	1·0	0·6, 1·4
University	0·9	0·6, 1·3
Household income^§^		
< 30 000 $CAD	1·1	0·7, 1·5
30 000 to < 60 000 $CAD	0·9	0·5, 1·2
60 000 to < 90 000 $CAD	0·8	0·5, 1·2
≥ 90 000 $CAD	1·0	0·6, 1·4
Smoking status		
Non-smokers	1·1	0·7, 1·4
Smokers	1·2	0·8, 1·6
Administrative region		
Capitale-Nationale/Chaudière-Appalaches	1·0	0·7, 1·4
Estrie	1·1	0·7, 1·5
Mauricie	1·2	0·8, 1·6
Montreal	1·0	0·7, 1·4
Saguenay-Lac St-Jean	1·2	0·7, 1·6

EAT-I, EAT-Lancet dietary index; CAD, Canadian dollars; CEGEP, Collège d’Enseignement Général et Professionnel.

* EAT-I scores calculated from estimated usual dietary intakes. Usual dietary intakes and costs estimated with the National Cancer Institute multivariate method.

† Diet cost differences comparing the 75th *v*. 25th percentiles of the EAT-I score distribution adjusted for total energy intake.

‡ 95 % CI estimated with 200 bootstrap resamples.

§ Missing sociodemographic characteristics imputed (see Methods section).

## Discussion

This study aimed to assess how a greater adherence to the EAT-Lancet reference diet impacts daily diet costs among adults from the province of Québec. Data collected between 2015 and 2016 suggest a positive association between the EAT-I score and energy-adjusted daily diet costs. In the overall population and among all sociodemographic groups investigated, the daily diet costs of those with a relatively high adherence to the EAT-Lancet reference diet were on average between 0·8 and 1·2 $CAD higher than the daily diet costs of those with a relatively low adherence to this reference diet. Greater adherence to the whole grains, vegetables, fruits, fish and plant-based proteins and free sugars components of the EAT-I were largely responsible for this increase in energy-adjusted daily diet costs. Inversely, greater adherence to the poultry and eggs component of the EAT-I was negatively associated with energy-adjusted daily diet costs.

Few studies have assessed the extent to which adhering to the EAT-Lancet reference diet influences the daily costs of the diet. A global analysis has revealed that adhering to this dietary pattern was affordable in high-income countries, such as Canada, but not in low-income countries^(25)^. In Australia, the EAT-Lancet reference diet was shown to be less costly than the typical Australian diet basket^(37)^. On the other hand, a study conducted in Albania by Llanaj *et al.* found no association between diet costs and adherence to the EAT-Lancet dietary pattern^(38)^. Results from these studies are at odds with our data. Discrepancies among studies may be explained by various factors, including differences in study methodologies. For example, foods consumed outside the home were not considered in our analysis in contrast with the study conducted by Llanaj *et al.* Nevertheless, the increase in daily diet costs observed with a higher adherence to the EAT-Lancet reference diet in our study sample of French-speaking adults in the province of Québec is not surprising, considering that healthier dietary patterns have been shown in numerous studies to be more expensive than unhealthy dietary patterns. For example, many studies conducted in the UK^(17)^, the USA^(14,15)^ and Canada^(18)^ have found that energy-standardised dietary patterns that align with food-based dietary guidelines are associated with an increase in diet costs, with some or no difference between sociodemographic subgroups^(14,15,18)^. Specifically, healthier dietary patterns cost on average $1·48 USD/d and $1·54 USD/2000 kcal more than lower-quality dietary patterns, as observed in a 2013 meta-analysis^(12)^, which is fairly consistent with data from the present study.

Greater adherence to the vegetables and fruits components of the EAT-I was associated with higher usual diet costs, consistent with other studies having shown that these were the most expensive foods of the EAT-Lancet reference diet^(25)^. In addition, the association between daily diet costs and some of the EAT-I component scores is also consistent with the notion that nutrient-rich and low-energy density foods are more expensive per calorie than highly processed foods high in free sugars and fats such as refined grains, snacks and sweets^(19,21,39,40)^. A greater consumption of fish and plant-based protein foods in the present study was associated with higher diet costs, which may be surprising considering that diets rich in plant-based protein foods have been associated with no or minimal impact on diet costs^(41,42)^. However, data from our group using the same cohort have shown that individuals consuming more plant-based protein foods also tended to consume less affordable foods such as vegetables and fruits^(41)^. Legumes, nuts and fish have also been found to account for an important share of the cost of the EAT-Lancet reference diet^(25)^, which is consistent with the present findings.

Having focused primarily on human health and benefits for the environment^(24)^, the EAT-Lancet Commission has been criticised for not having given due consideration to the economic and socio-cultural domains of diet sustainability, as well as for not meeting the needs of certain segments of the population for some nutrients^(43)^. Yet, economic accessibility is a key dimension of diet sustainability, and price is one of the main determinants of food choices for consumers^(26,27)^. The present findings, by documenting the economic constraints of adhering to the EAT-Lancet reference diet, confirmed the importance of considering the economic aspect of diets to facilitate the transition towards healthier and more sustainable dietary patterns. This further emphasises the importance of deploying food pricing strategies and policies such as subsidies, discounts and cash rebates on key sustainable foods to make healthy food choices more affordable^(44)^. Other promising approaches include strategies that discourage the purchase of unhealthy foods such as sugar-sweetened beverage taxes^(45)^. These strategies and policies will be of even greater importance in coming years with the escalating food prices in Canada and elsewhere^(46,47)^, a context that is likely to deteriorate further with the negative predicted effects of climate change on food prices and affordability^(48,49)^.

The present study has important strengths, including the sex and age representativeness of adults aged 18–65 years from five administrative regions of the province of Québec. The estimation of usual dietary intakes and daily diet costs using the NCI multivariate Markov Chain Monte Carlo method to account for within-individual random errors rather than relying on intake and cost data on a ‘given day’ is another key strength. We also argue that a continuous index reflecting adherence to the EAT-Lancet reference diet rather than categorical indices allowed a more refined analysis of its association with diet costs. The use of a food price database on actual prices paid by consumers in the province of Québec from 2015 to 2016 rather than food prices obtained from convenience samples is another strength. Limitations also need to be outlined. The Nielsen food price database used pertained to the fiscal year 2015–2016. Consequently, the associations observed are relevant to this specific period and may have changed due to more recent fluctuations in food prices in Canada due, among others, to global disruptions in food supply following the COVID-19 pandemic, the Ukraine’s war and erratic weather events among others^(50)^. Furthermore, the relatively high education and household income levels of the sample limit the generalisation of the results to other populations. In addition, we were not able to distinguish foods prepared at home *v*. commercial foods (e.g. pizza prepared at home *v*. commercial pizza). Food costs used do not represent the lowest price available at the time of purchase and were not available by type of store, season or geographic location. Foods and beverages reported were assumed to come from grocery or big box stores only, and foods consumed outside the home and food waste were not considered in the analysis. Finally, the EAT-I is influenced by the quality of the dietary intake data used, which are subjected to random and systematic errors despite best efforts to attenuate their impact.

In conclusion, the present study based on data collected between 2015 and 2016 suggests that, at any given amount of energy intake, a greater alignment with the EAT-Lancet Commission reference diet in the province of Québec is associated with greater daily diet costs. If supported by more recent diet cost analyses, these findings highlight the importance of considering the economic domain of diet sustainability when developing healthy and sustainable dietary patterns and guidelines. The present findings also emphasised the importance of making healthy and environmentally friendly food choices more affordable to face current challenges and to facilitate the transition towards more sustainable dietary patterns.

## Supporting information

Rochefort et al. supplementary material Rochefort et al. supplementary material

## Supplementary material

For supplementary material/s referred to in this article, please visit https://doi.org/10.1017/S0007114525000364.
