# Supplementary material for: Increased costs associated with greater adherence to the EAT-Lancet Commission reference diet in the province of Québec: the PREDISE Study
Source: Br J Nutr. 2025 Feb 27;133(6):837–44. doi: 10.1017/S0007114525000364 (PMC12169952; doi:10.1017/S0007114525000364)
Supplement: Rochefort et al. supplementary material [file S0007114525000364sup001.docx]

**SUPPLEMENTARY MATERIAL**

**Supplementary Table 1.** Methods used to calculate a price for each of the Bureau of Nutritional Science (BNS) food group

| Method used to compute a price | Bureau of Nutritional Science food groups |
| --- | --- |
| Prices obtained from the Nielsen database | 01A- Pasta  01B-Rice  02A- White bread  03A- Whole wheat breads  03B- Other whole grain breads  04A- Rolls, bagels, pita bread, croutons, dumplings, matzo, tortilla  04B- Crackers and crispbreads  04C- Muffins and english muffins  04D- Pancakes and waffles  04E- Croissants, piecrusts & phyllo dough  05A- Whole grain, oats and high fiber breakfast cereals  06A- Breakfast cereal (other)  07A- Cookies, commercial  07C- Granola bar  08A- Pies, commercial  08C- Danishes, doughnuts and other pastries, commercial  09A- Ice cream  09B- Ice milk  09C- Frozen yoghurt  10A- Milk, whole  10B- Milk, 2%  10C- Milk, 1%  10D- Milk, skim  10I- Other types of milk (whey, buttermilk)  10J- Plant-based beverage (soy, almond, coconut)  14- Cheese, unknown fat %  14B- Cheese, less than 10% b.f.  14C- Cheese, 10% b.f. to 25% b.f.  14D- Cheese, more than 25% b.f.  15- Yoghurts, unknown fat %  15A- Yoghurts, less than 2% b.f.  15B- Yoghurts, more than 2.1% b.f.  16A- Egg  22A- Beef, lean only  22B- Beef, lean + fat  22C- Beef, ground  23A- Veal, lean only  23B- Veal, lean + fat (incl ground veal)  25A- Pork, fresh, lean only  25B- Pork, fresh, lean + fat (incl ground pork)  25D- Ham, cured, lean only  25E- Ham, cured, lean + fat  27- Poultry, unknown type  27A- Chicken, meat only  27B- Chicken, meat + skin  27C- Turkey, meat only  28A- Liver  28B- Liver pate  30A- Sausage  32A- Luncheon meat  33A- Nuts  33B- Seeds  33C- Peanut butter and other nut spreads  34A- Fish, less than 6% total fat  34B- Fish, superior or equal to 6% total fat  35A- Shellfish  36- Vegetables, unknown type  36A- Beans  36B- Brocoli  36C- Cabbage and kale  36D- Cauliflower  36E- Carrots  36F- Celery  36G- Corn  36H- Lettuces & leafy greens (spinach, mustard greens, etc.)  36I- Mushrooms  36J- Onion, green onions, leeks, garlic  36K- Peas and snow peas  36L- Peppers, red & green  36M- Squashes  36N- Tomatoes  36O- Juices, tomato & vegetable  36P- Other veg (cucumber, immature beans, brussel sprouts, beets, turnips)  37A- Legume  37B- Foods made with vegetable proteins (tofu)  38A- Potato chips  38B- Fried or roasted potatoes  39A- Potato  40A- Citrus fruits (oranges, grapefruits, lemons, etc.)  40B- Apple  40C- Banana  40D- Cherries  40E- Grapes and raisins  40F- Melons (canteloup, honeydew, watermelon)  40G- Peaches, nectarines  40H- Pears  40I- Pineapple  40J- Plums and prunes  40K- Strawberries  40L- Other fruits (blueberries, dates, kiwis, fruit salads, etc.)  42A- Popcorn, plain & pretzels  42B- Salty and high-fat snacks (incl tortilla chips)  43A- Candies, gums, etc.  43B- Ice pop, sherbert  44A- Chocolate bar  45A- Fruit juice  46AB- Soft drink - unknown sugar content  46A- Soft drinks - regular  46B- Soft drinks – diet  46C- Fruit drinks  46D- Other beverages (malted milk, chocolate beverage)  46E- Energy drink  46F- Vitamin water  50A- Soups with vegetables  50B- Soups without vegetables  50C- Gravies  54A- Energy bar  54B- Protein bar and shake  140C- Other breads, crackers, rolls, dumplings, bannocks, bagels, english muffins  140E- Pancakes & waffles  150A- Cakes, cheesecakes, shortcakes and brownies  150B- Cookie  150C- Danishes, turnovers & pastries  150D- Donuts  150E- Muffins  150F- Pies (including pie shell)  150H- Sweet rolls and breads  202B- Ice milk (recipe sub-group)  203A- Yoghurt (recipe sub-group)  204A- Cheese (recipe sub-group)  210- Meat dishes, unknown type  211A- Chicken (recipe sub-group)  213A- Sausage, with cured and luncheon meat (recipe sub-group)  215A- Chinese (recipe sub-group)  216A- Mexican dish  217A- Legume dishes without meat  219F- Hotdogs  220B- French fries and hash brown potatoes  220F- Vegetables (mixed with other stuff)  226A- Nuts and seeds (recipe sub-group)  229A- Sweet snacks, sugar, candies (recipe sub-group)  230B- Soup, dehydrated  231D- Milk-based beverage (milk shakes, malted milk, hot cocoa, instant breakfast, etc.)  2223- Beef and veal, lean + fat |
| Prices obtained using data from the Government of Canada ^(1)^ | 01C-Cereal grains and flours;  10F-Milk, evaporated, 2%;  10G-Milk, evaporated, skim;  17A-Butter;  18A-Regular tub margarine;  18B-Calorie-reduced tub margarine;  21A-Vegetable oils;  41A-Sugars (white and brown);  50D-Sauces (white, bearnaise, soya, tartar, ketchup, etc.);  51A-Tea (including iced tea)  51B-Coffee; |
| Prices obtained using data from the Government of Quebec ^(2)^ | 41C-Other sugars (syrups, molasses, honey, etc); |
| The price of a comparable food from the Nielsen database, the Government of Canada, or the Government of Quebec was used with the application of a factor if necessary. | 10K-Goat and sheep milk;  13A-Whipping cream;  13B-Table cream;  13C- Half & half cream;  13D-Sour cream;  14A-Cottage cheese;  21B-Animal fats;  21C-Shortening;  24A-Lamb, lean only;  24B-Lamb, lean + fat (incl ground lamb);  25C-Bacon;  27E-Other birds (duck, pheasant, pigeon);  29A-Offal;  41B-Jams, jellies and marmalade;  41D-Sugar substitutes;  43C-Gelatin, dessert toppings and pudding mixes, commercial;  46G-Sports drink;  50E-Salad dressings (with or without oil);  54C-Meal replacements;  130F-Rice (recipe sub-group);  205A-Milk dessert (recipe sub-group);  212A-Fish, with less than 6% fat before cooking (recipe sub-group);  212C-Shellfish (recipe sub-group);  231J-Protein powder; |
| Prices obtained from the 2016 annual report of the Société des alcools du Québec ^(3)^ | 47A-Spirits;  47B-Liqueurs;  48A-Wine;  49A-Beer;  231G-Alcoholic beverages; |
| BNS food groups with missing price | 31A-Game meat;  50F-Seasonings (salt, vinegar, etc.);  51C-Water (well & mineral);  52A-Babyfood product;  53A-Spices;  53B-Others (baking soda, baking powder, yeast, etc.); |

**Supplementary Table 2.** EAT-I components and subcomponents, points, and scoring standards.^1^

| **Component or subcomponent** | **Measurement** | **Maximum points** | **Standard for minimum score** | **Standard for maximum score** |
| --- | --- | --- | --- | --- |
| 1. **Whole grains** | Ratio: Total whole-grain foods / Energy | 10 | 0%E | ≥ 32.4%E |
| 1. **Tubers and starchy vegetables** | Ratio: Total tubers and starchy vegetables/ Energy | 10 | ≥3.1%E | 0%E |
| 1. **Vegetables** | Ratio: Total non-starchy vegetables/ Energy | 10 | 0%E | ≥3.1%E |
| 1. **Fruits** | Ratio: Total fruits/ Energy | 10 | 0%E | ≥5.0%E |
| 1. **Dairy foods** | Ratio: Total dairy foods/ Energy | 5 | ≥12.2%E | ≤6.1%E |
| 1. **Red and processed meats** | Ratio: Total red and processed meats/ Energy | 5 | ≥2.4%E | 0%E |
| 1. **Poultry and eggs** | | | | |
| **Poultry** | Ratio: Total poultry and other birds/ Energy | 2.5 | ≥4.0%E | ≤2.5%E |
| **Eggs** | Ratio: Total eggs/ Energy | 2.5 | ≥1.5%E | ≤0.8%E |
| 1. **Fish and plant-based proteins** | | | | |
| **Fish and seafood** | Ratio: Total fishes and seafood/ Energy | 1.67 | 0%E | ≥1.6%E |
| **Legumes and soy** | Ratio: Total legumes and soy foods/ Energy | 1.67 | 0%E | ≥11.4%E |
| **Nuts and seeds** | Ratio: Total nuts and seeds/ Energy | 1.67 | 0%E | ≥11.6% |
| 1. **Added fats** | | | | |
| **Unsaturated added fats** | Ratio: Total unsaturated oils/ Energy | 5 | 0%E | ≥14.2% |
| **Saturated added fats** | Ratio: Total saturated oils and added fats/ Energy | 5 | ≥3.8%E | 0%E |
| 1. **Free sugars** | Ratio: Total free sugars/ Energy | 10 | ≥4.8%E | 0%E |

EAT-I, EAT-Lancet Dietary Index; E, Energy.

^1^ Table adapted from Rochefort et al. 2024 ^(4)^.

**REFERENCES**

1. Gouvernement du Canada & Statistique Canada (2021) Prix de détail moyens mensuels pour les aliments et autres produits sélectionnés. https://www150.statcan.gc.ca/t1/tbl1/fr/tv.action?pid=1810000201 (accessed January 2022).

2. Institut de la statistique du Québec (2020) Répartition du prix moyen de vente du miel selon le marché, par regroupement de régions administratives, Québec. https://statistique.quebec.ca/fr/produit/tableau/repartition-du-prix-moyen-de-vente-du-miel-selon-le-marche-par-regroupement-de-regions-administratives-quebec (accessed January 2022).

3. SAQ (2016) Rapport annuel 2016.

4. Rochefort G, Robitaille J, Lemieux S, et al. (2024) Are the 2019 Canada’s Food Guide Recommendations on Healthy Food Choices Consistent with the EAT-Lancet Reference Diet from Sustainable Food Systems? *J Nutr*, S0022-3166(24)00098–1.
